# Mycofiltration of Aqueous Iron (III) and Imidacloprid Solutions, and the Effects of the Filtrates on Selected Biomarkers of the Freshwater Snail *Helisoma duryi*

**DOI:** 10.1007/s00244-023-01049-3

**Published:** 2024-02-08

**Authors:** Sanele M. Mnkandla, Mafereka Francis Tyson Mosoabisane, Norah Basopo, Patricks Voua Otomo

**Affiliations:** 1 Ecotoxicology Research Group, Department of Zoology and Entomology, Faculty of Natural and Agricultural Sciences, University of the Free State, Qwaqwa Campus, Private Bag x13, Phuthaditjhaba, 9866 South Africa; 2 Ecotoxicology Research Group, Department of Applied Biology and Biochemistry, National University of Science and Technology, Bulawayo, Zimbabwe; 3 Afromontane Research Unit, University of the Free State, Private Bag x13, Phuthaditjhaba, 9866 Qwaqwa South Africa; 4 Department of Chemistry, Faculty of Natural and Agricultural Sciences, University of the Free State, Qwaqwa Campus, Private Bag x13, Phuthaditjhaba, 9866 South Africa; 5 Radiochemistry, South African Nuclear Energy Corporation Limited, Brits, 0240 South Africa

## Abstract

To alleviate the burden of water contamination, a newly developed form of bioremediation known as mycofiltration can be employed. Mycofiltration is an environment-friendly technology involving the treatment of contaminated water by passing it through a network of saprophytic fungal mycelium. A mycofilter made of *Pleurotus ostreatus* was used for the removal of iron (III) and imidacloprid from aqueous solutions. Batch mycofiltration, at a dosage of 1 g of mycofilter per 50 mL, was performed on iron (III) solutions of different concentrations (0.99, 10.7, 22.9, and 27.72 mg/L) and pH (3.3, 7 and 11). For column mycofiltration, the mycofilter was packed into pyrex columns (3.3 × 15 cm) to desired bed heights. Iron (III) and imidacloprid solutions of 18.99 mg/L and 234.70 ng/L, respectively, were filtered at a constant flow rate. Thereafter, *Helisoma duryi* snails were exposed for 96 h to the respective filtrates, and their catalase and acetylcholinesterase activities were assessed. Batch mycofiltration showed iron (III) removal rates as high as 85%. Column mycofiltration showed removal rates of 94 and 31% for iron (III) and imidacloprid, respectively. Catalase activity was significantly reduced (*p* < 0.05) in the snails exposed to iron (III) or imidacloprid filtrates, compared to the snails exposed to the non-mycofiltered media. A significantly higher acetylcholinesterase activity was induced by iron (III) filtrates in comparison with the non-mycofiltered media (*p* < 0.05). There were no significant differences in acetylcholinesterase activity (*p* > 0.05) in the snails exposed to mycofiltered and non-mycofiltered imidacloprid media. Mycofilter characterisation using Fourier Transform Infrared Spectrophotometry revealed significant changes in transmittance intensity in the mycofilters used for the iron (III) vs the ones used for the imidacloprid solutions. Mycofiltration was found to improve water quality although iron (III) was removed more effectively than imidacloprid.

Water is an essential natural resource which plays an important role in the provision of health, ecosystem services and environmental sustainability (Nkiaka et al. 2021). One of the targets under the United Nations Sustainable Development Goal 6 is the protection and restoration of water-related ecosystems by 2020 (https://www.un.org/sustainabledevelopment/water-and-sanitation/ [Accessed on 10 May 2023]). Achieving this target, however, tends to be compromised by anthropogenic activities such as farming, mining, poor water treatment and wastewater management, as they result in chemical insults to water bodies, threatening the health of the ecosystem. Pollution events due to heavy metals and pesticides are commonly occurring examples of such insults.

Heavy metals are defined as a group of metallic elements that have densities higher than that of water (Fergusson 1990). The heavy metal iron is an essential micronutrient present abundantly in the earth’s crust, but it is also present in increasing concentrations through environmental contamination (Sevcikova et al. 2011; Tchounwou et al. 2012). Iron exists in two oxidation states, the ferrous [Fe (II)] and ferric [Fe (III)] forms. In aqueous solutions, iron is prone to hydrolysis, forming hydroxides in the ferric form which have a low solubility. The pH of the solution influences the retention of iron in solution. In natural waters where the pH ranges between 5 and 8, iron is precipitated and exists as ferric hydroxide, causing water discolouration seen by a red/brown colour (Hem and Cropper 1962). In surface waters, the precipitates cause turbidity, affecting the amount of light that can penetrate to support aquatic life (Prasad and Danso-Amoako 2014). Ferric iron precipitates also accumulate on substrates on which aquatic organisms feed, causing physical stress and chemical toxicity in some organisms (Cadmus et al. 2018).

With the quest for improved agricultural productivity, pesticides such as imidacloprid are used. Imidacloprid falls under a class of pesticides known as neonicotinoids, which were introduced to the market as a new generation of environmentally safe insecticides (Cossi et al. 2020). Neonicotinoids act on postsynaptic nicotinic receptors present in the central nervous system of invertebrates, binding irreversibly and causing continuous transmission of the nerve impulse, leading to neural death (Ghanim and Ishaaya 2011). Imidacloprid is typically used for treating seeds, such that as the crop grows, the active part of the insecticide spreads to all parts of the plant tissue (Van Dijk et al. 2013; Simon-Delso et al. 2014). It is reported, however, that only 1.6–20% of the insecticide coated on the seed enters the crop to protect it, and the rest ends up in the environment, accumulating in the soil and leaching into ground or surface water (Van Dijk et al. 2013). In a study investigating the occurrence of pesticides in streams draining into agricultural watershed, it was found that imidacloprid exceeded environmental quality standards by 58-fold (Curchod et al. 2020). Due to its high water solubility, imidacloprid can affect non-target aquatic organisms, causing unintended toxic effects including endocrine disruption and mortality (Shan et al. 2020; Santiago et al. 2023).

To alleviate the contaminant burden present in water bodies, various bioremediation technologies can be employed. Bioremediation is said to be continually evolving, and an example of this is a recently developed form of mycoremediation known as mycofiltration (Taylor et al. 2015; Barbato and Reynolds 2021; Mnkandla and Otomo 2021). The “myco” prefix refers to fungi, which are eukaryotic organisms, existing as long chains of cells aggregated into mats, i.e., mycelia (Sankaran et al. 2010). Mycofiltration thus is the treatment of contaminated water by filtering it through a fungal mycelium network (Taylor et al. 2015). The mycelia employed are saprophytic in nature, i.e., they grow and feed on dead organic matter; thus, a typical mycofilter will be comprised of a mix of organic matter (i.e., substrate) and saprophytic mycelium (Stamets 2005). Fungi of the *Pleurotus* genus (oyster mushroom) have been used in mycofiltration. Under laboratory conditions, mycofilters of the *Pleurotus ostreatus* strain have shown to be resilient after being subjected to biological stresses such as cycles of freezing, drying, and heating, which are representative of conditions seen in the environment (Stamets et al. 2013). After going through these stresses, in one case study, the mycofilters significantly removed suspended *E.coli* (Stamets et al. 2013). These findings attest to the adaptable nature of fungi and their ability to thrive under hostile environmental conditions, thus making them very useful for bioremediation (Hamba and Tamiru 2016).

Fungal mycelia are said to employ mechanisms such as bioaccumulation, biodegradation and biosorption during the remediation process (Singh 2006; Kulshreshtha et al. 2014). Bioaccumulation is an active metabolism-dependent process, which involves transporting pollutants into the cells and partitioning them into intracellular components (Kulshreshtha et al. 2014). The biodegradation mechanism entails the degradation of non-polymeric and recalcitrant pollutants to simpler elements by extracellular enzymes (Kulshreshtha et al. 2014). Biosorption is a passive process which occurs on the cell surface by ion exchange and complexation reactions with functional groups such as carboxyl, amine, hydroxyl and phosphate (Bishnoi and Garima 2005). Studies have shown that the *Pleurotus* species, either live or dried biomass, have a very effective biosorption potential for metals such as Cu, Zn, Fe and Mn (Boamponsem et al. 2013). They have also demonstrated the ability to breakdown pesticides (Golan-Rozen et al. 2011; Camacho and Sánchez 2015).

Bioremediation serves to reduce the levels of chemical insults which could adversely affect life in, e.g., water bodies. It thus becomes important to investigate any biological changes, i.e., biomarkers, that may occur in organisms that are exposed to an environmental agent (Peakall 1994; Valavanidis et al. 2006). Sentinel animals or bioindicators are typically used to measure biomarker responses following exposure. These organisms’ occurrence and behaviour are closely correlated with defined ecological factors which serve as direct indicators of the changes or stresses in the ecosystem (Abdel-Halim 2018). Measurement of target enzymes is commonly used as biomarkers. Examples of these enzymes include antioxidant enzymes whose role is to counter the oxidative stress induced by contaminants, and esterases such as acetylcholinesterase which is considered a biomarker of neurotoxicity (Kaviraj et al. 2014; Rouibi et al. 2016; Abdel-Halim 2018).

Mycofiltration of iron has been described, where drastic reductions in iron levels in borehole and river water were observed (Akpaj and Olorunfemi 2014; Olorunfemi et al. 2015, 2020). In these studies, however, only the total iron content was assessed. Regarding the mycofiltration of pesticides, there is sparse information available. Against this backdrop, the purpose of the present study was to conduct mycofiltration of ferric iron and imidacloprid using the *P. ostreatus* mycelium. Furthermore, the quality of mycofiltered water was assessed through exposure studies using freshwater snails and assessment of catalase and acetylcholinesterase activities. Previous authors did not subject mycofiltered samples to exposure and biomarker studies; thus, this study aimed to cover this gap.

## Materials and Methods

### Preparation of Mycofilters

Mycofilters were prepared as described by Stamets and Chilton (1983) and Stamets et al. (2013), with slight modifications as outlined by Mnkandla and Otomo (2023). Briefly, thatching straw was collected from a veld near the University of the Free State, Qwaqwa, South Africa, and shred into small particles (1–2 mm) using a herb grinder, prior to soaking overnight in tap water containing 2% (w/v) calcitic lime. Thereafter, the water was drained, and the straw was pasteurised at 72 °C for 30 min, then allowed to cool. The working area was sterilised with 70% ethanol, left to dry, and wiped down with 2% (w/v) calcitic lime. The pasteurised straw was inoculated with *Pleurotus ostreatus* spawn purchased from a local supplier (Sylvan Africa (Pty) Ltd), at a spawning rate of 10% (w/w). The inoculated straw was then packed into transparent 10 × 12 cm polyethylene bags. The bags were securely tied, and eight small perforations were randomly made around the bags to allow for gaseous exchange, prior to being positioned spaciously in 4 L (25 cm × 17 cm × 8 cm) plastic containers. To ensure ideal conditions for the spawn run, the 4 L containers housing the bags were placed in a 30 L (52 cm × 35 cm × 20 cm) plastic tote. The tote was wrapped with aluminium foil (to ensure darkness) and filled with 600 mL tap water enough to cover the base (to ensure humidity). The bags were incubated at room temperature for 4–5 weeks or until 100% colonisation was visually observed.

### Preparation of Imidacloprid and Iron (III) Solutions

#### Imidacloprid Solution

A stock solution of 1 mg/L imidacloprid was prepared by dissolving 0.001 g imidacloprid PESTANAL® analytical standard in 1 L distilled water. To prepare the working solution, the stock solution was diluted with distilled water to a final concentration of 234.70 ng/L, at pH = 7.5. The concentration was confirmed by the accredited commercial pesticide analysis laboratory of the Tanzania Plant Health and Pesticides Authority, Arusha, Tanzania. The method they used was the Quechers En for analysis of pesticide residue on water effluent with TSQ LC-MS/MS triple quadrobe. Limits of detection and quantification were 0.2 and 0.5 µg/kg, respectively. Imidacloprid solutions were covered with aluminium foil to protect them from light and stored at 4 °C prior to use.

#### Iron (III) Solution

A stock solution of iron (III) was prepared by dissolving 1 g of ferric chloride in 100 mL distilled water. From the stock solution, 27.72, 22.9, 10.70 and 0.99 mg/L were prepared by dissolving with the required volume of distilled water. Solution pH was adjusted to 7 or 11 using 0.1 M NaOH.

**Fig. 1:**
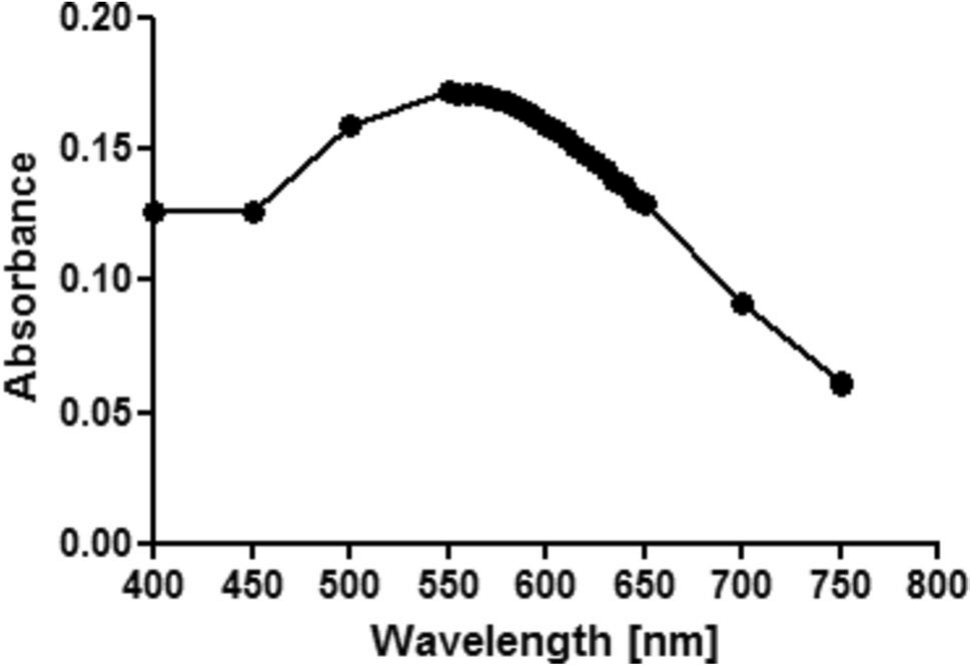
Visible spectrum of the Indian Gooseberry extract and its complex with iron (III) in acetate buffer at pH 5.6

Concentrations of iron (III) solutions were confirmed using the Indian gooseberry extract (tea powder) and spectrophotometry, as described by Rattanakit and Maungchang (2019). The extract forms a complex with iron (III) which can be detected spectrophotometrically. The extract was prepared by placing a small bag of the tea powder (1 g) in 150 mL distilled water and left to stand for 10 min at room temperature. The solution was stirred until mixed well, prior to removing the bag. For spectrophotometric determination, the following were mixed: 3 mL of the dissolved extract, 3 mL of 0.2 M acetate buffer (pH 5.6), an appropriate volume of iron (III) stock (for the standard solutions: 0–25 mg/L) and distilled water, to a final volume of 10 mL. The mixture was left to stand for 10 min. To select the wavelength for absorbance measurements, the maximum wavelength of the iron (III)-extract complex was determined. This was done by recording absorbances of the highest concentration of the iron (III) standards (i.e., 25 mg/L) over the range of 400–750 nm, using the visible V-10 Plus ONDA spectrophotometer. The maximum wavelength was found to be 550 nm (Fig. 1). Each standard was then measured at 550 nm, and the standard curve was plotted. All iron (III) solutions were mixed as described in this section and measured at 550 nm. The concentrations were determined from the standard curve.

#### Batch Mycofiltration of Iron (III)

To obtain the optimal solution pH and iron (III) concentration for removal by mycofiltration, batch mode experiments were performed. First, 50 mL aqueous solutions with varying pH = 3.3, 7 and 11 and each containing 27.72 mg/L iron (III) were prepared in 250 mL Erlenmeyer flasks. Then, 50 mL aqueous solutions with a fixed pH = 7 and varying iron (III) concentrations = 27.72, 22.9, 10.70 and 0.99 mg/L were prepared in 250 mL Erlenmeyer flasks. In each of the flasks, 1 g of the *P. ostreatus* mycofilter was added. The flasks were placed on a platform shaker (FMH instruments) at 100 rpm for 120 min. About 2 mL of iron (III) solution were taken at different time intervals (15, 30, 60 and 120 min) and quantified spectrophotometrically as described in the previous section.

#### Column Mycofiltration of Iron (III) and Imidacloprid

Mycofilter columns were prepared, in duplicate, by packing the colonised straw from the polyethylene bags in Pyrex columns (3.3 cm internal diameter and 15 cm height) to yield a desired bed height (5.3 cm or 8 cm). At room temperature, imidacloprid (234.70 ng/L, pH 7.5) or iron (III) (18.99 mg/L, pH 7) solutions were passed through the columns in a downflow mode at 0.45 mL/min, using a peristaltic pump (SP-Minipump 022 [dual channel]). Effluent samples were collected at the bottom of the column at varying time intervals: 0, 0.3, 2, 4, 8, 16, 24, 48, 72 and 120 h. Iron (III) concentrations were measured as described in the previous section. Imidacloprid was quantified as previously mentioned.

Plots of C/C_0_ versus time, i.e., breakthrough curves (where *C*_0_ is the inlet concentration and *C* is outlet or effluent concentration at the time intervals) were drawn for iron (III) and imidacloprid. Removal efficiency was calculated as:

$${\text{Removal efficiency }} = \, \left[ {\left( {C_{0} - C} \right)/C_{0} } \right] \, \times \, 100$$

Mycofiltered iron (III) and imidacloprid media were stored at 4 °C prior to exposure studies.

#### Fourier Transform Infrared Spectrophotometry (FTIR) Analysis

To investigate mycofilter functional groups, FTIR analysis was performed. In duplicate, 4 g of used mycofilter, i.e., after the filtration of iron (III) or imidacloprid, and unused mycofilter (control) were placed in separate beakers and air dried under a fume hood, for 72 h. Samples were then crushed to powder using a pestle and mortar and sieved through a 250 µm pore size sieve. Analysis was performed using the PerkinElmer Spectrum 100 FTIR spectrophotometer attached with a PIKE Miracle™ Attenuated Total Reflectance (ATR) and equipped with a diamond crystal. Each sample was gently placed on the spot of the FTIR accessory and slowly pressed. Spectra were recorded in the 4000–650 cm^−1^ with 4 cm^−1^ resolution range, accumulating 8 scans.

#### Snail Breeding, Exposures, and Biomarker Response Analysis

Freshwater snail species, *Helisoma duryi,* were bred in 50 L polypropylene tanks containing tap water and were fed on fresh garden lettuce twice a week as described by Naik and Hasler (2002). Prior to exposures, the snails were acclimatised to the laboratory conditions by being placed in tap water for 3 days. The number of snails used for exposures in this study was guided by Shuhaimi-Othman et al. (2012). In duplicate, groups of 4 snails were placed in 350 mL of iron (III) or imidacloprid solutions. The snails were exposed to 18.99 mg/L iron (III)/ and 234.70 ng/L imidacloprid, respectively, before and after mycofiltration. The concentration of ion (III) and imidacloprid in the respective filtrates was subsequently analysed as mentioned above. The control group was exposed to tap water only. The exposures were performed for 96 h at room temperature, and the snails were fed with lettuce ad libitum.

After the exposures, the snails were frozen and homogenised in 3 volumes of ice cold 0.1 M potassium phosphate buffer, pH 7. The homogenates were centrifuged at 10,000×*g* for 15 min at 4 °C, and the post-mitochondrial fraction (PMF) was collected. Protein determination was carried out using the BCA Protein Colorimetric assay kit (Elabscience), according to the manufacturer’s instructions. Absorbances were measured at 562 nm using the Genova Nano spectrophotometer. Total protein concentration in the PMF samples was obtained from the standard curve.

Catalase activity was assayed as described by Claiborne (1985). Briefly, 2900 μL of 19 mM hydrogen peroxide solution in 50 mM potassium phosphate buffer (pH 7) were gently mixed with 100 μL of sample (1 mg/mL PMF) and the rate of decrease in absorption was monitored at 240 nm for 30 s, using the Genova Nano spectrophotometer. Specific activity was expressed as units/mg protein. Acetylcholinesterase activity was measured according to the method described by Ellman et al. (1961). The reaction mixture contained 550 μL of 0.01 M Tris/HCl buffer pH 8.0, 250 μL of 1 mg/mL PMF and 250 μL of 10 mM Ellman’s reagent (5, 5 dithio-bis-(2 nitro benzoic acid)). The mixture was incubated at 37 °C for 3 min, before adding 150 μL of 30 mM acetylthiocholine iodide. The rate of increase in absorption was measured at 412 nm, for 5 min, using the Jenway 7300 spectrophotometer. Specific activity was expressed as μmol/min/mg protein.

### Statistical Analysis

The analysis of enzyme activities was reported as means ± SD. A one-way analysis of variance (ANOVA) with multiple comparisons (Turkey’s multiple comparisons test) was used to analyse the effect of exposure media (i.e., non-mycofiltered, mycofiltered or tap water media) on enzyme activity. Statistical analysis and graph plots were done using GraphPad Prism 6 (GraphPad Inc, San Diego, USA). Significance of results was ascertained at *p* < 0.05.

## Results

**Fig. 2:**
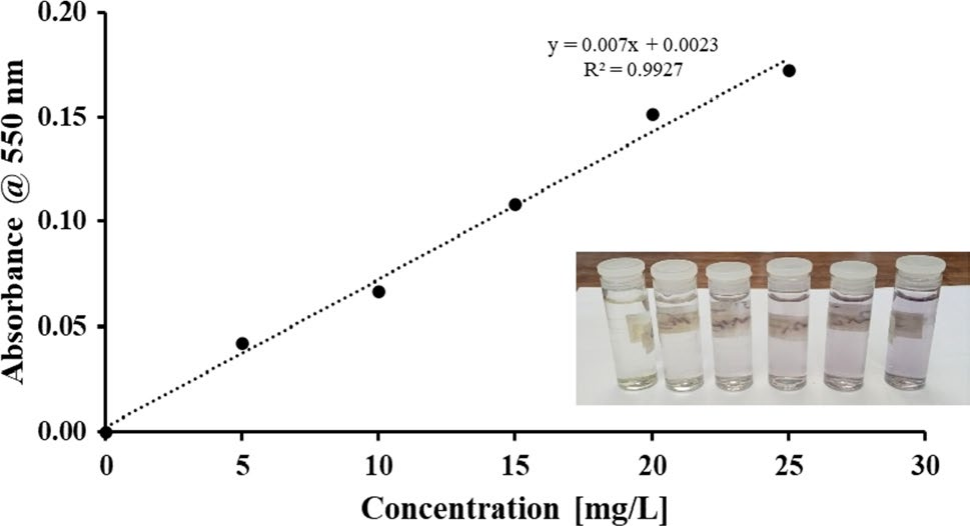
Standard curve for Iron (III) determination using Indian Gooseberry extract and spectrophotometry. Insert: Image showing colour change in the solution containing iron (III) at concentrations of (from left to right) 0, 5, 10, 15, 20 and 25 mg/L

Figure 2 shows the standard curve for iron (III) standards quantified using the Indian gooseberry extract. The insert shows the colour change progression as the iron (III) concentration increased. Colour change is a result of the reaction between gallic acid present in the extract, and iron (III) forming an iron-polyphenol complex (Rattanakit and Maungchang 2019).

**Fig. 3:**
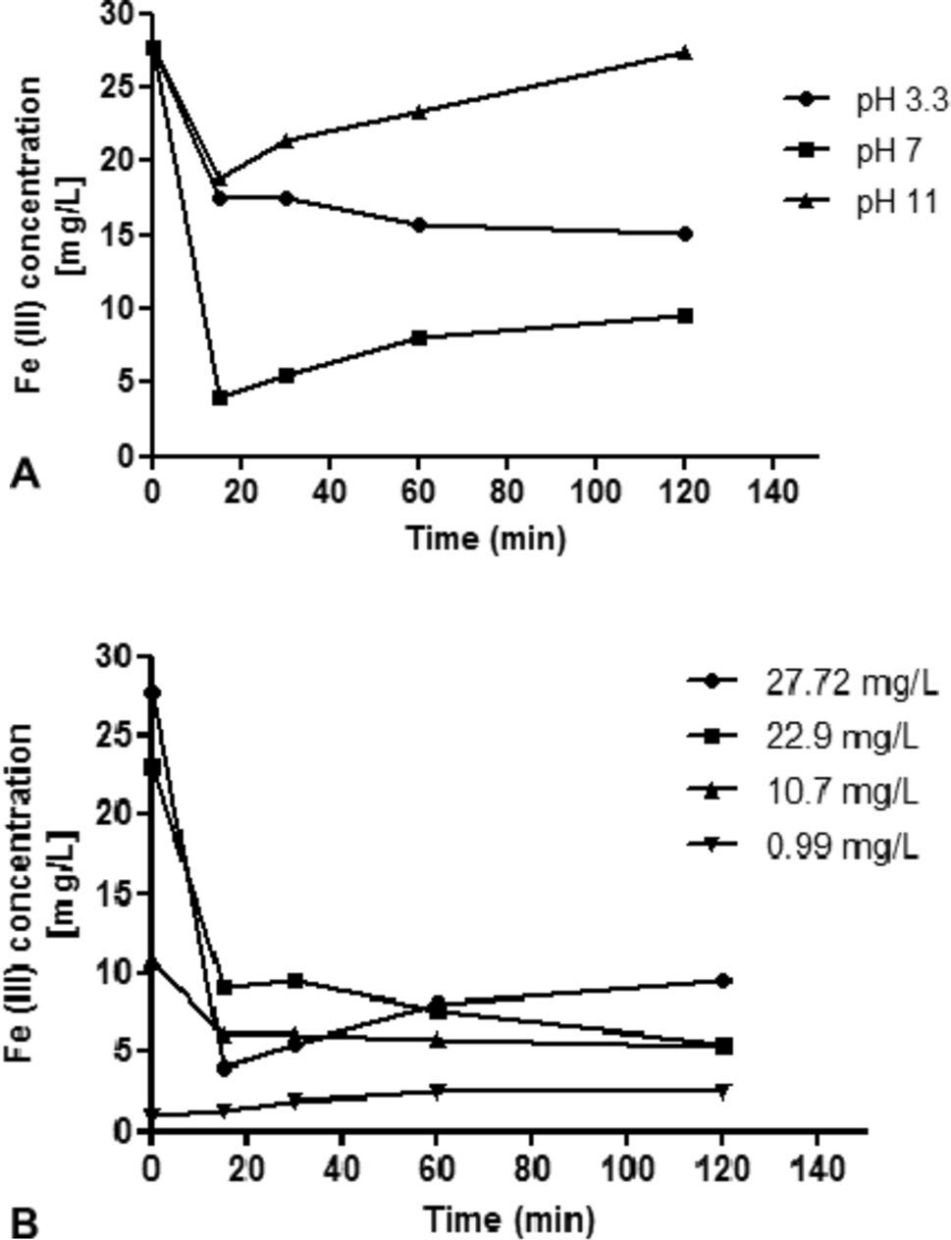
Batch mycofiltration experiments varying **A** solution pH, and **B** Initial iron (III) concentration. *Pleurotus ostreatus* mycofilter (1 g) was placed in 50 mL solution and kept on a rotary shaker at 100 rpm for 120 min. In varying pH conditions, the solution concentration was 27.72 mg/L, and in varying iron (III) concentration conditions, the solution pH was 7

Batch mycofiltration of iron (III) is shown in Fig. 3. When the concentration was kept at 27.72 mg/L iron (III) and the solution pH was 3.3, 7 or 11, at 15 min of mycofiltration, the greatest biosorption was observed at pH = 7, compared to pH = 3.3 and 11 (Fig. 3A). There was 85% biosorption at pH = 7 and only 37 and 33% biosorption when pH was 3.3 and 11, respectively (Fig. 3A). After 120 min contact time, 65, 45 and 1% biosorptions were observed at pH = 7, 3.3 and 11, respectively (Fig. 3A).

When solution pH was kept at 7, and iron (III) concentration varied from 0.99, 10.70, 22.90, and 27.72 mg/L, biosorption was more efficient at higher initial concentrations such as 22.90, and 27.72 mg/L (Fig. 3B). After 120 min contact time, there was 65, 75 and 51% biosorption where the initial iron (III) concentrations were 27.72, 22.90 and 10.70 mg/L, respectively (Fig. 3B). There was no biosorption observed where iron (III) concentration was 0.99 mg/L and after 120 min exposure time, iron (III) levels had increased to 2.4 mg/L (Fig. 3B).

**Fig. 4:**
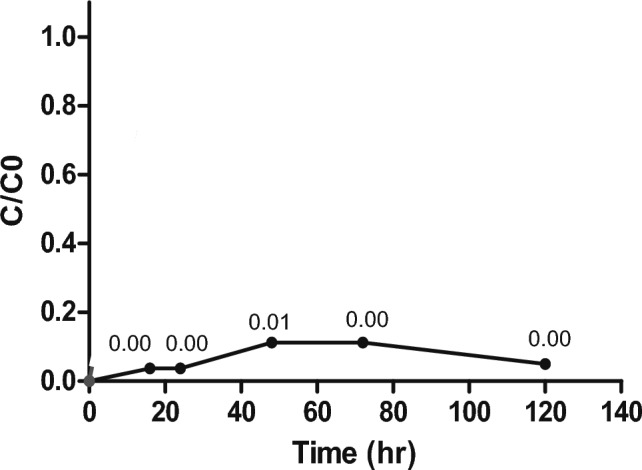
Column mycofiltration of iron (III) solution. Iron (III) concentration was 18.99 mg/L and solution pH = 7. Column was operated at a flow rate of 0.45 mL/min with a *Pleurotus ostreatus* mycofilter (adsorbent) bed height of 8 cm. Values are means ± SD of two replicates

Figure 4 shows the column mycofiltration of iron (III). Mycofiltration was run at a constant flow rate of 0.45 mL/min, and the solution concentration and pH were 18.99 mg/L and 7, respectively. The biosorption of iron (III) appeared to be very effective, as there was 94% removal at the end of 120 h (Fig. 4). The concentration of iron (III) in the filtrate was 0.99 mg/L. Interestingly, the column did not reach saturation as the curve remained well below the *C*/*C*_0_ value of 1 (Fig. 4).

**Fig. 5:**
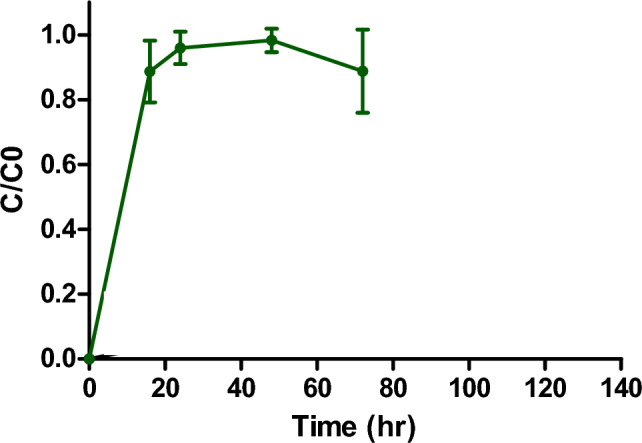
Column mycofiltration of Imidacloprid solution. Imidacloprid concentration was 234.70 ng/L and solution pH = 7. Column was operated at a flow rate of 0.45 mL/min with a *Pleurotus ostreatus* mycofilter (adsorbent) bed height of 5.3 cm. Values are means ± SD of two replicates

Column mycofiltration of imidacloprid at a concentration of 234.70 ng/L, solution pH = 7 and flow rate of 0.45 mL/min is shown in Fig. 5. The column saturated early at 24 h, shown by the curve reaching a *C*/*C*_0_ value of 0.9 (Fig. 5). Only 31% of imidacloprid was removed after 72 h (Fig. 5). Imidacloprid concentration in the filtrate was 187.00 ng/L.

**Fig. 6:**
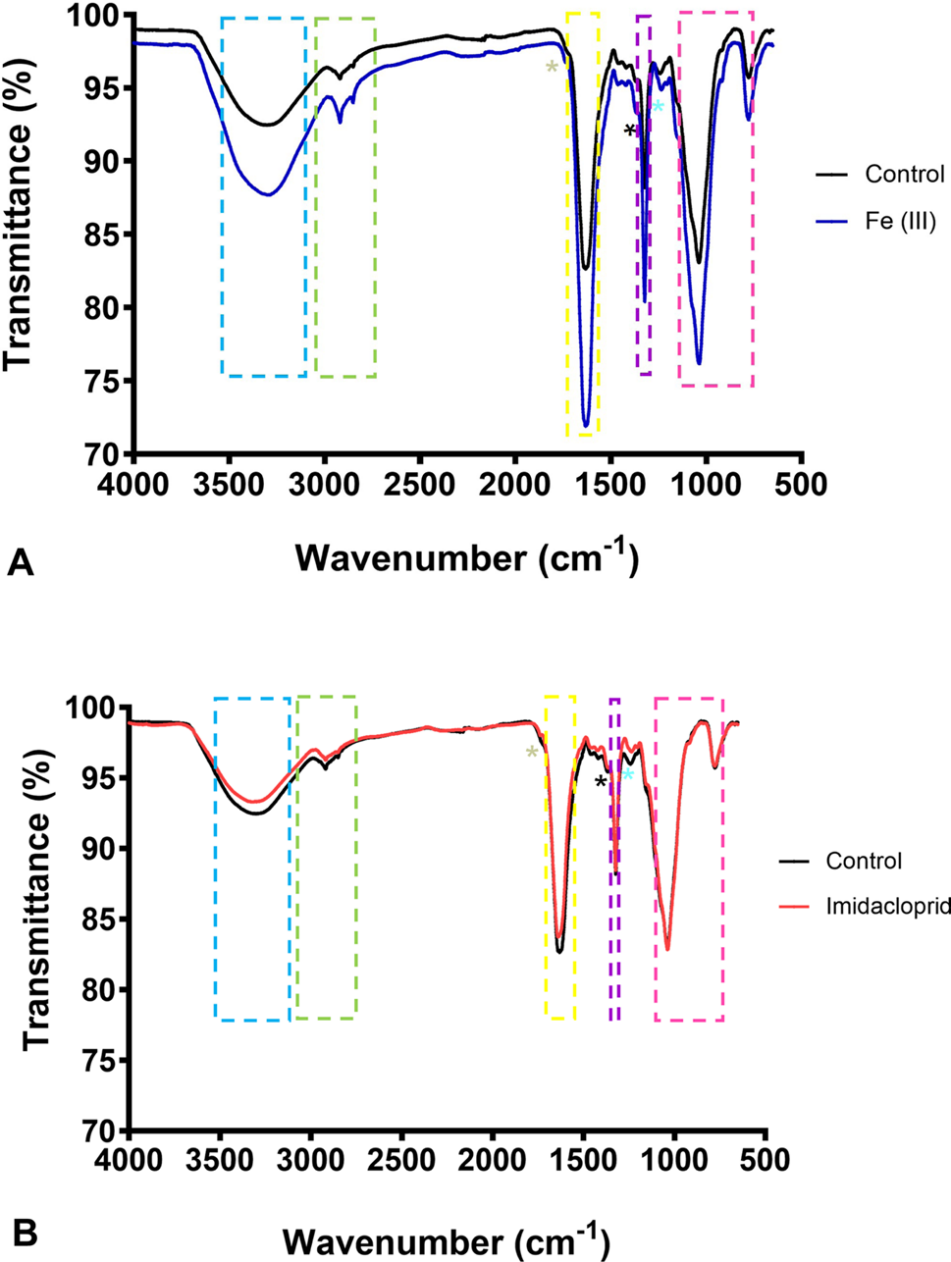
FTIR spectra for *Pleurotus ostreatus* mycofilters. A comparison of **A** the control mycofilter (i.e., no filtration) versus the mycofilter which filtered iron (III) solution, and **B** the control mycofilter (i.e., no filtration) versus the mycofilter which filtered the imidacloprid solution. The coloured dashed boxes represent the functional groups. Blue and magenta: polysaccharides, green: lipids, yellow and purple: protein (amide groups), gold: overlapping shoulder for ester bonds, black asterisk: chitin, turquoise asterisk: phosphate group

The FTIR spectra of the control mycofilters vs spent mycofilters are shown in Fig. 6. Compared to the control mycofilter, there were notable compositional changes in the mycofilter used for the mycofiltration of iron (III) (Fig. 6A). There were increases in intensity (i.e., % Transmission) by the broad peak at 3306 cm^−1^, short peak at 2919 cm^−1^ and at the sharp narrow peaks at 1632 and 1034 cm^−1^ (Fig. 6A). In contrast, there were no significant differences observed between the control mycofilter and the mycofilters used for imidacloprid mycofiltration as the spectra were almost superimposed onto each other (Fig. 6B).

**Fig. 7:**
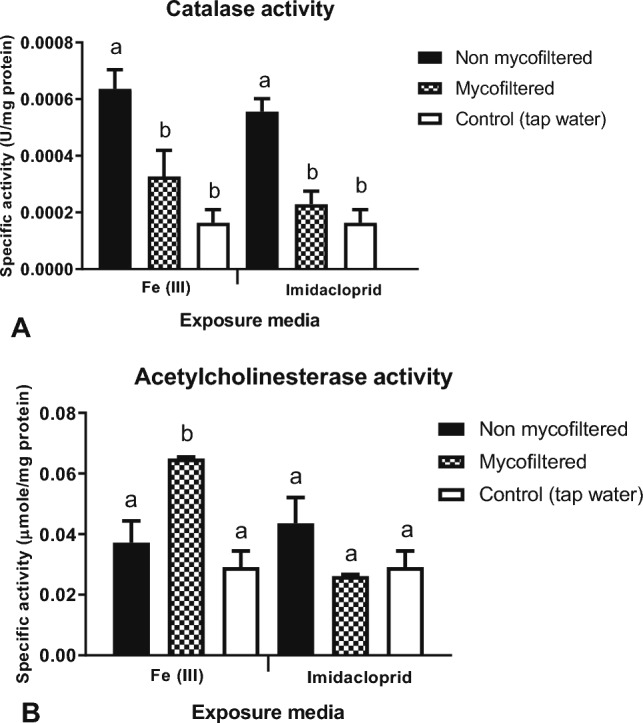
The effect of non-mycofiltered or mycofiltered iron (III) and imidacloprid on **A** catalase and **B** acetylcholinesterase enzyme activities of freshwater *Helisoma duryi* snails exposed for 96 h. All values are means ± SD of two replicates. Bars with different letters indicate significant differences (*p* < 0.05) within Fe (III) or imidacloprid exposure media

The effect of non-mycofiltered or mycofiltered media on catalase and acetylcholinesterase enzyme activities of *H. duryi* snails is presented in Fig. 7. Following 96 h of exposure, catalase activity was significantly higher (twofold) in the snails exposed to non-mycofiltered iron (III) compared to those exposed to mycofiltered iron (III) and tap water (Fig. 7A). Mycofiltration thus significantly reduced catalase activity from 0.000636 to 0.000327 U/mg protein (Fig. 7A). Catalase activity in the snails exposed to mycofiltered iron (III) was higher than that of the snails exposed to tap water (0.000163 U/mg protein), albeit insignificantly (Fig. 7A).

A similar trend in catalase activity was observed with the snails exposed to imidacloprid. The catalase activity in snails exposed to non-mycofiltered imidacloprid was 0.000556 U/mg protein and was significantly higher than the enzyme activity of 0.000229 U/mg protein of the snails exposed to mycofiltered imidacloprid and tap water (Fig. 7A). Relative to the control, there was no significant difference in catalase activity of the snails exposed to mycofiltered imidacloprid (Fig. 7A).

Regarding acetylcholinesterase activity, snails exposed to non-mycofiltered iron (III) had an activity of 0.032 µmol/min/mg protein and did not differ with the control (Fig. 7B). Interestingly, the enzyme activity in the snails exposed to mycofiltered iron (III) significantly increased by twofold, in comparison with the enzyme activity of those exposed to non-mycofiltered iron (III) and control tap water (Fig. 7B).

With regards to imidacloprid exposures, there were no significant differences in acetylcholinesterase activities of snails exposed to non-mycofiltered or mycofiltered imidacloprid or tap water (Fig. 7B). Enzyme activities ranged between 0.025 and 0.037 µmol/min/mg protein (Fig. 7B).

## Discussion

Regarding batch mycofiltration of iron (III) [Fig. 3A], where solution pH was above 3.3, the ferric ions precipitated as ferric hydroxides. At higher pH, iron (III) precipitates because of the high concentrations of hydroxyl ions in aqueous media (Ahalya et al. 2006). In the present study, biosorption was nonetheless still possible at a higher pH of 7, plausibly due to fungal processes of metal solubilisation. For growth and metabolism, fungi use organic matter as their source of energy and secrete organic acids such as citric acid and oxalic acid (Gadd et al. 2014). The acidic metabolites then solubilise solid matrix metals by forming soluble metal complexes and chelate ions (Xia et al. 2018). Fungi also secrete ferric iron chelating compounds known as siderophores, whose function is to acquire iron from insoluble hydroxides or from iron adsorbed to solid surfaces (Winkelmann 2002; Haas 2003). It is reported that the optimum pH conditions for siderophore production range between pH = 7–7.5 (Patel et al. 2017; Srivastava et al. 2022). These siderophores, with a high affinity for ferric iron, form ferric-siderophore complexes which then follow specific uptake mechanisms for recovery by the fungal cells (Winkelmann 2002). Oxalic acid and siderophores work synergistically in their interaction with iron (Dehner et al. 2010), thus allowing for biosorption to take place.

Recent work done in our laboratory (Mnkandla and Otomo 2023) found that the pH point of zero charge (i.e., the pH value where the surface density of positive charges equals that of negative charges) of the *P. ostreatus* mycofilter was 6.5; thus, any pH above this would result in the surface charge of the mycofilter being negatively charged, allowing for the binding of positively charged cations. In this instance where pH = 7, iron (III) could biosorb on the mycofilter. Menaga and colleagues (2021), working with iron (II), also observed complete biosorption at solution pH = 7, using *Pleurotus florida* spent mushroom substrate. They attributed their findings to the ionisation states of the functional groups of the fungal cell wall, where the negatively charged groups were potent scavengers of the iron cations. The low biosorption observed at pH = 3.3 in the present study could thus be due to repulsion between the iron cation and the positively charged surface of the mycofilter.

Pertaining to batch mycofiltration of iron (III), when pH was kept at 7 and the concentration varied from 0.99 to 27.72 mg/L (Fig. 3B), more biosorption was observed as the concentration increased from 10.70 to 22.90 mg/L. At the highest concentration of 27.72 mg/L, biosorption decreased. Our findings are similar to those of Zareh and co-workers (2022), who observed increased biosorption of iron (III) as the concentration increased from 10 to 22 mg/L, but thereafter any increase in iron (III) did not result in more biosorption. They ascribed this to the proportion of free active adsorbent binding sites and initial number of iron (III) ions. At a lower iron (III) concentration, there are more binding sites available for biosorption. In the highest concentration, free binding sites are fewer (as most are occupied) leading to a decrease in biosorption. In our study, however, biosorption did not occur with the lowest iron (III) concentration of 0.99 mg/L, possibly due to a low driving force that could not overcome mass transfer resistances of the iron (III) between the aqueous and solid phases. Higher concentrations thus boost uptake capacity (Kamarudzaman et al. 2013). It is not clear why the iron (III) concentration increased from 0.99 to 2.40 mg/L. Perhaps there was leaching of metal from the straw component of the mycofilter.

Fixed-bed columns are evaluated by breakthrough curves which show the pollutant-effluent concentration at a fixed-bed's outlet as a function of time (Barros et al. 2013). The biosorbate is quickly biosorbed by the top biosorbent layers as soon as the influent enters the column through the inlet, leaving little to no biosorbate in the resulting effluent (Patel 2019). As biosorption progresses, the breakthrough point is reached, i.e., when the biosorbate concentration in the effluent is 10% of its initial concentration (Barros et al. 2013; Patel 2019). As the column saturates, the biosorbate concentration in the effluent increases, until no biosorption takes place. At this point, the ratio of outlet concentration/inlet concentration (*C*/*C*_0_) is 1 (Patel 2019).

In the present study, fixed-bed column biosorption of iron (III) [Fig. 4] showed that the column conditions were optimum, as the iron (III) solution had sufficient time to interact with the mycofilter for maximum biosorption. Not only did the column not reach breakthrough point, but it never saturated after running for 120 h, suggesting that there was room to filter more iron (III) solution. Unfortunately, the same could not be said for the fixed-bed mycofiltration of imidacloprid, where the column was saturated in 24 h (Fig. 5). Mandal and colleagues (2017), who investigated different agro-waste products for the sorption of imidacloprid, observed that sorption capacity is influenced by a variety of factors such as polarity of the sorbate, aromaticity, surface functional groups and specific surface area. Of the five products they tested, only the physicochemical properties of eucalyptus bark showed significant correlation with sorption of imidacloprid. It is therefore possible that the *P. ostreatus* mycofilter, used in the present study, did not possess properties suitable for the optimum sorption of imidacloprid. This is also evidenced by the FTIR spectra of the mycofilter after filtration of imidacloprid (Fig. 6B), where no significant differences were found when compared to the control spectra, suggesting very little sorption.

Spectral comparison of the control vs iron (III) mycofilter shows significant compositional changes in the iron (III) mycofilter (Fig. 6A), suggestion biosorption. The peaks at 3306 cm^−1^, 2919 cm^−1^, 1632 cm^−1^ and 1034 cm^−1^ with increased % transmission correspond to hydroxyl, lipid, amide and polysaccharide groups, respectively. These functional groups are provided for by the components that make up the mycelial cell wall (Haneef et al. 2017). Of the listed components, the polysaccharides play a significant role as they complex with iron (III) to enhance iron absorption. The alcoholic hydroxyl group of polysaccharides is deprotonated and coordinated to iron (III) (Somsook et al. 2005). Our findings are like those of Liu and colleagues (2019) who synthesised a polysaccharide-iron (III) complex in the edible saprophytic fungi *Astragalus membranaceus* and observed notable changes in the 2000–800 cm^−1^ spectra range, indicating complexation of iron ions.

In the present study, the mycofiltration intervention showed that water quality was improved to some extent, as evidenced by the biomarker responses of the snails exposed to the media. It has been reported in some field studies that aquatic life is not affected at iron concentrations above the water quality criterion of 1000 µg/L (Loeffelman et al. 1985; Ohio EPA 1998). However, single species toxicity tests performed by Cadmus and colleagues (2018), where they exposed a taxonomically diverse group of aquatic organisms to ferric chloride at various concentrations, revealed that iron was sub-lethal, reducing growth, development, and reproduction. In our study, *H. duryi* snails exposed to non-mycofiltered iron (III) media showed increased catalase activity (Fig. 7A), indicating that the organisms were under oxidative stress which induced the antioxidant protective response. Catalase is considered as the first line of defence against reactive oxygen species, and it acts by reducing hydrogen peroxide produced by the reaction of superoxide dismutase acting on superoxide radicals (Matés and Sánchez-Jiménez 2000). Similarly, the snails exposed to non-mycofiltered imidacloprid media were under oxidative stress evidenced by increased catalase activity (Fig. 7A). After mycofiltration, iron (III) and imidacloprid levels were reduced, and the subsequent catalase response in the exposed snails significantly decreased.

Acetylcholinesterase is the enzyme responsible for the breakdown of the neurotransmitter acetylcholine, terminating synaptic transmission thus preventing continuous nerve firings at nerve endings (Lionetto et al. 2013). Acetylcholinesterase activity is a biomarker of neurotoxicity, where alteration in activity indicates chemical toxicity (Basopo et al. 2014). Acetylcholinesterase activity of the snails exposed to mycofiltered iron (III) in the present study (Fig. 7B) was significantly higher than that of the snails exposed to non-mycofiltered media. Pohanka (2014), who was working on human acetylcholinesterase, observed the significant potency of iron (III) in the inhibition of acetylcholinesterase. It was shown that the mechanism of inhibition was non-competitive, with the metal binding to the anionic site of the acetylcholinesterase catalytic canter. This could possibly explain the suppressed acetylcholinesterase activity of the snails exposed to non-mycofiltered media in this study, as the iron (III) concentration was high. After mycofiltration, the iron (III) concentration was insufficient to cause major inhibition.

Another possible explanation for the increased acetylcholinesterase activity of the snails exposed to mycofiltered iron (III) in this study could be the secretions by the mycelia which were present in the mycofiltered media. As mentioned earlier, oxalic acid is secreted by mycelia upon interaction with iron (III). Oxalic acid itself also acts as an acaricide and is reported to potentially alter acetylcholinesterase activity in honeybees (Rouibi et al. 2016). Perhaps the iron-oxalic acid complexes which may not have biosorbed onto the mycofilter were present in the mycofiltered media and were bioaccumulated by the snails, and interfered with acetylcholine breakdown, subsequently causing increased acetylcholinesterase activity. Concerning imidacloprid, snails exposed to either the mycofiltered or non-mycofiltered media did not show significant differences in acetylcholinesterase activity (Fig. 7B). Our findings are similar to those of Cossi et al (2020) who observed that cholinesterase activity in the gastropod *Biomphalaria straminea* was not affected by the neonicotinoid acetamiprid. In their study, they concluded that neonicotinoids bind specifically to the nicotinic acetylcholine receptors; therefore, effects on the acetylcholinesterase enzyme are not usually expected.

## Conclusion

The *P. ostreatus* mycofilter demonstrated selectivity in the type of sorbent it can effectively remove. Iron (III) was more favoured compared to imidacloprid. Efficient mycofiltration of iron (III) is, however, concentration dependent, where there is more removal with higher concentrations. The mycofilter column not reaching saturation after running for 5 days is a positive indicator for potential field application, as it is ideal to have a filter which can run efficiently for long periods before the need for a replacement. The mycofilter operating optimally at pH = 7 also points to the potential of use practically in water bodies with circum-neutral pH. Biomarker analysis showed that mycofiltration does improve water quality, shown by lowered catalase enzyme activities in *H. duryi* snails exposed to mycofiltered iron (III) or imidacloprid. In some instances, however, possible products formed due to interactions of the biosorbate and live mycelia may affect exposed organisms, as observed by the increased acetylcholinesterase activity in the snails exposed to mycofiltered iron (III) in this study.

## Data Availability

The datasets generated during and/or analysed during the current study are available from the corresponding author on reasonable request.
